# Altered superoxide dismutase-1 activity and intercellular adhesion molecule 1 (ICAM-1) levels in patients with type 2 diabetes mellitus

**DOI:** 10.1371/journal.pone.0216256

**Published:** 2019-05-01

**Authors:** Anderson Martins Tavares, Jaslana Hainfellner Silva, Christiane de Oliveira Bensusan, Andrea Claudia Freitas Ferreira, Livia Pinto de Lima Matos, Kleber Luiz de Araujo e Souza, Luciene de Carvalho Cardoso-Weide, Giselle Fernandes Taboada

**Affiliations:** 1 Programa de pós graduação em Ciências Médicas, Universidade Federal Fluminense, Niterói, RJ, Brasil; 2 Escola de Biologia, Universidade Salgado de Oliveira (UNIVERSO), Niterói, RJ, Brasil; 3 Instituto de Biofísica Carlos Chagas Filho, Universidade Federal do Rio de Janeiro, Rio de Janeiro, RJ, Brasil; 4 Multidisciplinary Center for Biological Research (Numpex-Bio), Universidade Federal do Rio de Janeiro, Duque de Caxias, RJ, Brasil; 5 Departamento de Ciências Básicas, Universidade Federal Fluminense, Nova Friburgo, RJ, Brasil; 6 Departamento de Patologia, Faculdade de Medicina, Universidade Federal Fluminense, Niterói, RJ, Brasil; 7 Departamento de Medicina Clínica, Faculdade de Medicina, Universidade Federal Fluminense, Niterói, RJ, Brasil; 8 Faculdade de Medicina, Universidade Estácio de Sá, Rio de Janeiro, RJ, Brasil; National Institutes of Health, UNITED STATES

## Abstract

Inflammation and oxidative stress are linked to type 2 diabetes mellitus (T2DM). In this work, we analyzed patients’ blood markers of antioxidant capacity, oxidative stress and inflammation in individuals with T2DM, in pre-diabetes state (pre-DM) and controls without diabetes. Patients were divided into three groups, according to glycated hemoglobin A1c (HbA1c): <7%, 7–9%, and >9%. Superoxide dismutase (SOD) and glutathione peroxidase (GPX) activities, total thiols, nitric oxide (^•^NO), tumor necrosis factor alpha (TNF-α) and intercellular adhesion molecule 1 (ICAM-1) levels of the individuals were measured. Plasma SOD activity was higher in T2DM subjects compared to the controls. While total thiols levels were lower in T2DM groups when compared to pre-DM, the values remained unchanged when compared to controls. ICAM-1 levels of T2DM groups were lower than in controls, while GPx activity, ^•^NO, and TNF-α levels were similar among all groups. A positive correlation was found between SOD and HbA1c levels. Concluding, individuals with T2DM present altered SOD activity, total thiols, and ICAM-1 levels, which might contribute to further complications. There is a positive correlation between SOD activity and HbA1c levels. No apparent correlation exists between total thiols and ICAM-1 levels and with any other of the parameters evaluated in this study.

## Introduction

Diabetes mellitus (DM) is a metabolic disorder characterized by chronic hyperglycemia due to impaired insulin production from pancreatic beta cells and/or insulin resistance [1]. Chronic hyperglycemia is linked to oxidative stress, which involves increased reactive oxygen species (ROS) production. Antioxidant defense systems may be impaired and cellular/tissue damage may ultimately result. The antioxidant defense systems comprise enzymatic and non-enzymatic mechanisms, which ensure the balance between ROS production and scavenging. The enzymatic antioxidant scavenging system includes enzymes, particularly superoxide dismutase (SOD) and glutathione peroxidase (GPx), while the non-enzymatic system involves circulating thiols (SH) [2, 3]. Increased glycated hemoglobin levels (HbA1c) measured in the serum correlates with development and severity of micro and macrovascular complications and this might reflect the overall redox status of T2DM patients [4–8]. Moreover, there is evidence suggesting that reduced nitric oxide (^•^NO) levels result in endothelial dysfunction [9] and that oxidative stress induces nuclear factor kappa B (NFκB) pathway resulting in an increase of pro-inflammatory cytokines, such as tumor necrosis factor alfa (TNF-α) as well as intercellular adhesion molecule 1 (ICAM-1) [10].

Strict glycemic control in type 2 diabetes mellitus (TDM2) is important to prevent DM complications. Hyperglycemia may be associated with increased SOD and GPx activity, as well as total thiols levels, to compensate for ROS generation. Moreover, it may influence circulating inflammatory cytokines (ICAM-1 and TNF-α) and serum ^•^NO levels. To test this hypothesis, we sought to evaluate oxidative stress and inflammatory markers in patients with pre-DM and TDM2 stratified according to HbA1c levels compared to controls without DM. Our results provide evidence for alterations in the (anti)oxidative profile of T2DM patients, but at the same time exclude most of correlations that could be surmised to a hyperglycemic profile, like correlations to HbA1c or to TNF-α levels.

## Patients and methods

### Patients

The study included 89 patients with T2DM categorized according to HbA1c % [estimated average glucose in nmol/mol] in three subgroups (Group A: HbA1c <7% [53]; Group B: HbA1c 7–9% [53–75]; Group C: HbA1c> 9% [75]), 12 patients with pre-diabetes (HbA1c between 5.7 and 6.4% [39 and 46]) and 12 control individuals (never diagnosed with DM nor pre-diabetes; HbA1c <5.7% [39]). Patients with and without microvascular complications (retinopathy determined through routine annual fundoscopy and/or nephropathy through annual microalbuminuria testing) were equally included to all T2DM groups to eliminate possible interference with the results. Exclusion criteria were: smoking, alcohol abuse, chronic viral diseases (hepatitis B and C or HIV), a severe infectious disease in the last 6 months and glomerular filtration rate below 60 mL/min, calculated with CKD-EPI formula [11]. Patient’s medical records were reviewed to obtain additional data for the study: presence of comorbidities such as systemic arterial hypertension and dyslipidemia, history of macrovascular disease and/or events (cardio or cerebrovascular event and / or non-traumatic limb amputation; previous diagnosis of coronary, carotid or peripheral arterial disease through a relevant complementary method), current medications as well as current anthropometric and laboratory data. The study was approved by the Ethics Committee of the Hospital Universitário Antônio Pedro of Universidade Federal Fluminense, Niterói, Rio de Janeiro, Brazil and written informed consent was obtained from each patient prior to study enrollment. Study and data collection ocurred between January 2016 and October 2018.

## Methods

### Collection and storage of samples

Blood samples were collected from fasting individuals into tubes, containing anticoagulant to obtain plasma or in tubes without anticoagulant to obtain serum. Tubes were centrifuged at 1000 rpm for 20 minutes to obtain the plasma or 3000 rpm for 15 minutes to obtain serum. Serum and plasma were aliquoted in cryotubes and stored at -80°C until further processing.

### Oxidative stress evaluation

#### Measurement of ^•^NO

Quantification of ^•^NO in serum was performed by fluorimetric assay (Cayman Chemical, Michigan, USA) after serum deproteinization, using a 30 kDa ultrafilter (Millipore, Massachusetts, USA) to obtain the ultrafiltrate. In this assay, total ^•^NO concentration of each sample was obtained from the reduction of nitrate (NaNO_3_) to nitrite (NaNO_2_), catalyzed by nitrate reductase. After constructing a NaNO_3_ standard curve, the regression coefficient and straight line equation were obtained. Total ^•^NO concentration (pmol/mL) was estimated from the emitted fluorescence of each sample by a fluorimeter—SpectraMax M3 Multi-Mode Microplate Reader (Molecular Devices, California, USA) at a wavelength of 375 nm excitation and 417 nm emission.

#### Plasma SOD and GPX activity

Antioxidant enzymes activities (SOD and GPx) were measured in plasma using commercial kits (Cayman Chemical, Michigan, USA). For SOD, one unit of SOD promoted 50% dismutation of ^•^O—(generated by xanthine oxidase) to H O and O The enzyme also reduces the tetrazolium salt, leading to the formation of red formazan. Plasma SOD activity (U/mL) was estimated by the absorbance from red formazan measured in a spectrophotometer- SpectraMax M3 Multi-Mode Microplate Reader (Molecular Devices, California, USA) at a 440 nm wavelength.

GPx activity (nmol/min/mL) was indirectly measured by the coupled reaction with glutathione reductase (GR) which oxidates NADPH. Oxidation of NADPH to NADP^+^ resulted in a decrease in absorbance obtained by a spectrophotometer at a 340nm wavelength.

#### Evaluation of total thiols

Total plasma thiols were measured using a colorimetric method described in [12] with modifications. Briefly, the 5,5'-dithiobis(2-nitrobenzoic acid) (DTNB) reacted with the thiolate anion of the plasma samples in thiol/disulfide exchange reaction, resulting in the formation of yellow NTB, which was read by absorbance at 412 nm (ε = 13.6 mM/ml, pH 8.0).

### Inflammatory profile

#### Quantification of ICAM-1 and TNF-α

Assessment of ICAM-1(ng/mL) and TNF-α (ng/mL) in plasma was performed using ELISA commercial kits (Sigma-Aldrich, Missouri, USA) in a spectrophotometer SpectraMax M3 Multi-Mode Microplate Reader (Molecular Devices, California, USA) at a 450 nm wavelength.

#### Statistical analysis

Data were analyzed using SPSS 23.0 for Windows (SPSS, Inc., Chicago, IL) and Graph Prism 6.0 for Windows (GraphPad Software, California, USA). Numerical variables were expressed as median [p25-p75]. The Kolmogorov-Smirnov test was performed to evaluate for normality of numerical variables. One-Way ANOVA or Kruskal-Wallis tests were used to compare numeric variables between multiple groups. Student's t-test and Mann-Whitney test were used to compare numeric variables between two groups. Correlations between numerical variables were evaluated using the Pearson or Spearman correlation coefficients. p values < 0.05 were considered statistically significant.

## Results

### Clinical and laboratory parameters

Clinical and laboratory parameters of the individuals are shown in Table 1. Drugs for T2DM in use were predominantly those provided by the Brazilian Public Health System which are: metformin (93,2%—n = 83/89), insulin (NPH and regular– 68,5%—n = 61/89) and sulfonylureas (glicazide or glibenclamide– 30,3%—n = 27/89). Insulin was used by 11 patients from group A, 22 patients from group B and 28 patients from group C. Sulfonylureas were used by 12 patients from group A, 13 patients from group B and 2 patients from group C. Groups were similar regarding age, DM duration, BMI, triglycerides (TG) and high-density lipoprotein (HDL-c). Serum levels of total cholesterol (TC) and LDL-c (mg/dL) were higher in controls when compared to pre-DM and DM groups. Among DM groups, TC and LDL-c were higher in group C when compared to groups A and B (Table 1).

**Table 1:** Clinical characterization and laboratory parameters of control, pre-DM and T2DM groups.

Parameters	Control (n = 12)	Pre-DM (n = 12)	Group A HbA1c < 7% (n = 28)	Group B HbA1c 7–9% (n = 32)	Group C HbA1c > 9% (n = 29)	p value*
**Sex (M/F)**	1/11	4/8	13/15	8/24	5/24	-
**Age (years)**	57 (50–73) n = 12	54 (49–67) n = 12	63 (56–66) n = 28	57 (51–62) n = 32	58 (49–63) n = 29	ns
**Duration of DM (months)**	-	-	120 (36–180) n = 28	126 (54–213) n = 32	134 (108–186) n = 29	ns
**BMI (kg/m²)**	-	-	29,6 (26,8–31,3) n = 27	32,3 (28–35,8) n = 32	29,5 (25,8–31,6) n = 29	ns
**FPG (mg/dL)**	91 (86–93) n = 6	89 (68–94) n = 9	104 (76–130) n = 28	150 ^a^^,^^b^^,^^c^ (123–173) n = 32	165 ^a^^,^^b^^,^^c^ (112–266) n = 29	<0,0001
**HbA1c** **(%; [nmol/mol])**	5,4 (5,2–5,5) n = 9	6,2 (6,1–6,3) n = 12	6,4 (6,0–6,7) n = 28	7,8 ^a^^,^^b^^,^^c^ (7,3–8,6) n = 32	11,1 ^a^^,^^b^^,^^c^^,^^d^ (9,7–11,6) n = 29	<0,0001
**TC (mg/dL)**	206,5 (179,8–234) n = 6	180 (156,5–227,8) n = 8	148 ^a^ (134–166) n = 28	169 (142,8–199) n = 32	183^c^ (157–224,5) n = 29	0,0045
**HDL-c (mg/dL)**	46,5 (41–72,7) n = 6	43 (32,7–49,5) n = 8	44 (38–51) n = 28	43,5 (37–51,75) n = 32	47 (38–59,5) n = 29	ns
**LDL-c (mg/dL)**	129,5 (104,5–158,3) n = 6	99 (78–148,3) n = 8	81 ^a^ (64–105) n = 28	96,5 (81–124,8) n = 32	117 ^c^ (87–154,5) n = 29	0,0068
**TG (mg/dL)**	99,5 (71–129,8) n = 6	183,5 (99,5–380,8) n = 8	96 (78–127) n = 28	111 (70–192,8) n = 32	88,5 (66,5–148,5) n = 28	ns

Results are represented as median (p25-p75).

^a^: <0.05 versus control

^b^: <0.05 versus pre-DM

^c^: <0.05 versus group A

^d^: <0.05 versus group B

*: p value for Kruskal-Wallis and One-Way ANOVA tests.

ns: not significant; Pre-DM: pre-diabetes; HbA1c: glycated hemoglobin; DM: diabetes mellitus; FPG: fasting plasma glucose; TC: total cholesterol; HDL-c: high density lipoprotein; LDL-c: low density lipoprotein; TG: triglycerides; M: male; F: female.

### Oxidative stress evaluation

Plasma SOD activity was higher in T2DM groups when compared to control (control: 1.67 [1.39–1.93] vs. group A: 2, 95 [2.22–3.55], p <0.001; vs. group B: 2.18 [1.97–3.34], p<0.01 and vs. group C: 2.38 [1.97–3.74]; p <0.01). SOD activity was also higher in T2DM group A when compared to pre-DM (pre-DM: 2.23 [1.56–2.74] vs. group A: 2.95 [2.22–3.55]; p <0.05). No difference was found in SOD activity neither between control and pre-DM groups nor between pre-DM and T2DM groups B and C (Table 2).

**Table 2:** Oxidative stress markers and inflammatory profile of control, pre-DM and T2DM groups.

Parameters	Control (n = 12)	Pre-DM (n = 12)	Group A HbA1c < 7% (n = 28)	Group B HbA1c 7–9% (n = 32)	Group C HbA1c > 9% (n = 29)	p value*
**SOD activity (U/mL)**	1,67 (1,39–1,93) n = 11	2,23 (1,56–2,74) n = 11	2,95 ^a^^,^^b^ (2,22–3,55) n = 27	2,18 ^a^ (1,97–3,34) n = 32	2,38 ^a^ (1,97–3,74) n = 28	0,004
**GPx activity (nmol/min/mL)**	80,9 (63,8–106,3) n = 10	83,5 (71,3–92,2) n = 11	77,4 (55,5–87,5) n = 28	73,8 (62,9–90,3) n = 32	74,3 (64,7–86,9) n = 29	ns
**Total thiols (mmol/mL)**	1,18 (1,13–1,12) n = 12	1,36 (1,14–1,97) n = 11	1,16 ^b^ (0,95–1,26) n = 25	1,16 ^b^ (0,97–1,46) n = 32	1,15 ^b^ (0,99–1,45) n = 28	0,01
**Total NO (pmol/μL)**	1,52 (0,54–4,56) n = 8	2,01 (0,79–3,04) n = 8	3,12 (0,45–16,1) n = 22	1,91 (0,72–10,3) n = 17	5,27 (1,15–10,3) n = 20	ns
**ICAM-1 (ng/mL)**	269,6 (184,2–335,3) n = 11	216,6 ^a^ (179,5–238) n = 12	197,8 ^a^ (167,3–228,8) n = 25	204,1 ^a^ (171,6–238,2) n = 32	206,3 ^a^ (165,4–228,3) n = 28	0,0021
**TNF-α (pg/mL)**	1,31 (0,69–2,25) n = 5	1,68 (0,79–2,01) n = 5	0,51 (0,26–1,42) n = 10	0,91 (0,32–1,38) n = 9	1,41 (0,83–1,86) n = 9	ns

Results are represented as median (p25-p75).

^a^: <0.05 versus control

^b^: <0.05 versus pre-DM.

*: p value for Kruskal-Wallis and One-Way ANOVA tests.

ns: not significant. SOD: superoxide dismutase; GPx: glutathione peroxidase; NO: nitric oxide; ICAM-1: intercellular adhesion molecule 1; TNF-α: tumor necrosis factor alpha; pre-DM: pre-diabetes; T2DM: type 2 diabetes mellitus; HbA1c: glycated hemoglobin.

Total thiols levels were lower in T2DM groups A, B and C, compared to pre-DM (pre-DM: 1.36 [1.14–1.97] vs. group A: 1.16 [0.95–1.26], p <0.05; vs. group B: 1.16 [0.97–1.46], p <0.05 and vs. group C: 1.15 [0.99–1.45], p <0.05). No difference was found in total thiols levels from control individuals compared to pre-DM or T2DM groups (Table 2).

Plasma GPx activity and Total ^•^NO were similar between groups (Table 2).

### Inflammatory profile

Plasma ICAM-1 levels were lower in T2DM groups when compared to control (control: 269.6 [184.2–335.3] vs. Group A: 197.8 [167.3–228.8], p <0.01, vs. group B: 204.1 [171.6–238.2], p <0.05 and vs. group C: 206.3 [165.4–228.3], p <0.05) (Table 2). No statistically significant difference was found in ICAM-1 levels between pre-DM and control nor T2DM groups. Also, plasma TNF-α levels were similar between groups (Table 2).

No differences were found in laboratorial, oxidative stress and inflammatory parameters between patients with and without microvascular complications. A moderate negative correlation was found between DM duration and plasma TNF-α levels (r = -0.43; p = 0.02; n = 28).

No other correlations were found among clinical, laboratorial, oxidative stress and inflammatory parameters.

## Discussion

DM is one of the major global public health challenges of the 21st century as the disease is associated with pathophysiological changes in various organs, including heart, kidneys and vascular system [13]. However, whether pre-DM precedes the development of diabetes complications is still a matter of debate. In order to test this hypothesis, the present study aimed to evaluate oxidative stress and inflammatory markers in control, pre-DM and T2DM patients stratified by their HbA1c.

A major contributor to the development of T2DM complications is oxidative stress, due to prolonged hyperglycemia and impaired oxidant/antioxidant balance [14]. The increase in plasma SOD activity of T2DM individuals found in our study is possibly an adaptive response to increased oxidative stress. In this scenario, SOD is important because it is the first line of defense against pro-oxidant molecules [2, 15]. Literature data on the behavior of plasma and intracellular SOD activity in T2DM patients are conflicting. When compared to healthy individuals, T2DM patients have shown increased [16, 17], decreased [18, 19] or equivalent [20, 21] SOD plasma or intracellular activity. Noteworthy on our results was the higher SOD activity in group A (i.e., T2DM subjects with HbA1c lower than 7%) compared to the other two groups of T2DM patients, suggesting decreased SOD activity as glycemic control worsens.

Another important enzymatic antioxidant defense is GPx, which promotes the degradation of H_2_O_2_ [16]. In our study, we expected to find an increase in GPx activity in T2DM groups paralleling the increased SOD activity. Nevertheless, we found no difference in GPx activity between T2DM, pre-DM and control groups. As for SOD, data regarding GPx activity in T2DM are also controversial: Plasma and intracellular GPx levels have been shown to be decreased [22, 23] or unchanged [16] in T2DM patients compared to healthy controls. Increased SOD activity unaccompanied by an increase in GPx could lead to increased H_2_O_2_ levels that could be deleterious, but measuring this is masked because of the apparent blood volume and the natural reactivity of H_2_O_2_.

Thiol groups of plasma proteins (mainly albumin) also participate in the non-enzymatic antioxidant system. They are highly nucleophilic and interact rapidly with free radicals helping to maintain the extracellular reducing ambient [24, 25]. The decreased thiol levels of T2DM patients compared to controls found in our study probably reflects the higher thiol consumption in an environment of increased oxidative stress. Our results are corroborated by previous data showing decreased thiol levels in T2DM patients [26–28], which have also been linked to the presence of diabetes complications [26, 27].

The levels of ^•^NO are important markers of nitrooxidative stress and reduced bioavailability of ^•^NO contributes to the pathogenesis of DM complications [9, 13, 29, 30]. Reported increased [31–33], decreased [18, 34] or not changed [35, 36] serum and plasma total ^•^NO levels in T2DM subjects compared to healthy subjects have been described. In our study, no significant difference in total serum ^•^NO levels among T2DM, pre-DM and control individuals were found. However, technical limitations for assaying ^•^NO might be interfering with the results, since median ^•^NO levels between groups are quite different, but there is a large dispersion of the individual values. Further investigations are deserved to clarify this issue.

Increased production of ROS leads to the release of pro-inflammatory cytokines, such as TNF-α, activating NFkB-related transcription of pro-inflammatory genes such as ICAM-1 [37]. Therefore, it is somehow surprising that our results showed no differences in TNF-α levels, since increased levels would be expected because of diabetes-related oxidative stress [38–40]. However, taking into consideration only the T2DM patients, there is a fall followed by a recover of the TNF-α levels as the glycemic control worsens. Therefore, it is possible that a link exist between glycemic control and inflammation, particularly, it is intriguing possibility that advanced glycation endproducts (AGEs) might interfere with TNF-α and/or global cytokine profile. The decrease in ICAM-1 levels is also unexpected result, since a positive correlation to TNF-α could be expected [37]. One possible explanation is that most of the T2DM patients of our study were using gliclazide and/or insulin, which have been described to reduce serum ICAM-1 levels in T2DM patients [41, 42].

Another unexpected result was the moderate negative correlation found between DM duration and plasma TNF-α levels. In fact, TNF-α levels has been shown to increase with longer duration of disease [43] as well as hyperglycemia [44] and to decrease with insulin and sulfonylurea treatment [44–47]. It is well known that disease duration is a contributor to poor glycemic control. In our patients disease duration and TNF-α levels seemed to increase through increasing HbA1c levels, however neither parameters did reach statistical significance. The small number of patients with measured TNF-α levels in each group and the preferential use of insulin over sulfonylurea in patients from higher HbA1c level (group C) might have interfered with these results.

## Conclusion

T2DM subjects present increase in SOD activity and lower levels of ICAM-1. The pre-DM subjects have increased total thiols levels compared to controls, a characteristic that is lost in T2DM subjects as the disease progresses. The increase in SOD activity suggests an adaptive response to increased oxidative stress in the T2DM. It seems that pre-DM individuals were not yet suffering from the deleterious effects of oxidative stress since higher total thiol levels might give an extra line of protection. Further studies are required in order to corroborate the role of glycemic control in the antioxidant and inflammatory profile of T2DM patients.

## Supporting information

S1 Table (PDF)
