# Supplementary material for: Altered superoxide dismutase-1 activity and intercellular adhesion molecule 1 (ICAM-1) levels in patients with type 2 diabetes mellitus
Source: PLoS One. 2019 May 1;14(5):e0216256. doi: 10.1371/journal.pone.0216256 (PMC6493748; doi:10.1371/journal.pone.0216256)
Supplement: S1 Table — (PDF) [file pone.0216256.s001.pdf]

Sexo	Idade	Etnia	DM	Retinopatia	Laser	Nefropatia	Complicações
2	53	1	36	0	0	0	0
1	63	1	120	1	0	0	1
2	65	2	192	0	0	0	0
1	61	0	180	0	0	0	0
2	75	2	144	1	0	1	1
1	60	2	264	0	0	0	0
2	68	0	12	0	0	0	0
1	56	0	144	1	1	1	1
1	47	1	168	0	0	0	0
1	54	0	48	1	0	0	1
1	64	0	120	1	1	0	1
2	50	1	22	0	0	0	0
1	49	0	24	0	0	0	0
2	44	0	36	0	0	1	1
1	69	0	300	0	0	1	1
1	67	1	96	0	0	0	0
1	71	0	120	1	0	0	1
2	62	2	45	0	0	0	0
2	64	1	108	0	0	0	0
1	65	0	84	0	0	0	0
1	63	2	120	1	0	0	1
2	64	1	120	0	0	0	0
2	73	1	420	1	1	0	1
2	56	1	24	0	0	0	0
2	58	2	24	0	0	0	0
2	70	1	348	0	0	0	0
2	63	2	300	1	1	1	1
2	56	1	192	1	0	0	1
1	47	1	17	0	0	0	0
1	51	0	252	1	1	1	1
2	49	2	48	0	0	0	0
2	68	1	228	1	1	0	1
2	56	1	252	1	0	1	1
2	57	0	108	0	0	0	0
2	54	2	106	0	0	0	0
2	65	1	204	0	0	1	1
2	51	0	24	1	0	1	1
2	55	1	168	1	1	0	1
2	59	2	252	1	0	0	1
2	51	0	7	0	0	0	0
2	57	1	168	0	0	0	0
1	57	1	35	1	0	0	1
1	50	0	132	1	0	1	1
2	66	1	216	1	0	0	1
1	56	0	156	1	0	1	1
2	69	2	276	0	0	0	0
2	66	2	132	0	0	0	0
2	60	1	72	0	0	0	0
2	49	0	48	0	0	0	0

1	59	2	45	0	0	0	0
2	58	0	252	0	0	0	0
2	62	1	72	0		0	0
2	62	0	132	0	0	0	0
1	51	2	96	0	0	0	0
2	67	0	240	0	0	0	0
1	65	0	120	1	0	0	1
2	57	0	72	0	0	1	1
2	57	1	96	1	1	0	1
2	59	0	48	0	0	1	1
2	61	2	144	0	0	0	0
2	60	1	120	1	0	0	1
2	54	1	120	1	0	0	1
2	53	2	204	0	0	0	0
2	46	0	132	1	1	0	1
2	58	1	122	0	0	1	1
2	55	2	134	1	0	1	1
2	67	2	156	0	0	0	0
2	64	2	288	1	0	0	1
1	70	1	264	1	0	1	1
2	56	0	180	1	0	1	1
2	46	1	106	1	0	0	1
2	63	0	252	0	0	1	1
2	60	2	156	1	0	1	1
1	66	0	300	0	1	0	0
2	58	0	156	0	0	0	0
2	59	1	60	0	0	0	0
2	44	2	204	0	0	0	0
2	49	2	1	0	0	0	0
1	53	1	84	1	1	1	1
2	41	0	108	0	0	0	0
2	60	0	180	1	1	1	1
2	42	0	168	1	0	0	1
2	48	1	108	0	0	1	1
1	66	0	2	0	0	0	0
2	66	0	120	0	0	0	0
1	58	0	108	0	0	0	0
2	32	2	36	0	0	0	0
2	79	2	192	1	1	0	1
2	74						
2	64						
2	49						
2	67						
1	60						
2	49						
2	72						
2	46						
1	54						
2	47						
1	51						

1	
2	57
2	51
2	80
2	48
2	49
2	57
2	83
2	57
2	71
2	42
1	47
2	61

GrupoEstudo	lacrovascul	HbA1c	ControleA1c	SOD	GPx	NO Total	ICAM-1	Tiol
1	0	6,8	1	1,93	52,21	29,80		
4	1	6,9	1	4,64	56,54	11,49		
1	0	6,8	1	15,53	104,17	13,51	204,98	1,186
1	0	6,8	1	19,00	93,73	8,13	403,04	1,486
4	1	6,2	1	15,65	52,47	37,58	169,19	1,489
1	0	6,4	1	3,37	6,88	26,22	167,58	0,946
1	0	6,8	1	3,01	87,11	7,13	196,68	1,010
4	0	5,5	1	3,19	6,62	23,70	149,29	1,199
1	0	6,5	1	2,45	51,96	3,54	162,35	1,316
4	0	6,8	1	1,66	114,10	0,25	221,13	1,213
4	1	6,7	1	3,39	76,92		208,12	1,032
1	0	6,5	1	2,22	87,61	0,10	178,25	1,559
1	0	6,5	1	1,70	112,06	1,88	232,98	1,176
4	1	6	1	3,36	96,78	1,07	254,21	0,841
4	0	5,5	1	1,87	77,94	2,71	246,84	1,267
1	1	6	1	2,59	44,32	0,27	210,56	0,909
4	0	6,5	1	1,79	79,97		183,13	1,228
1	1	6,1	1	3,60	65,71		255,40	0,870
1	0	6,1	1	2,90	65,71	0,09	156,87	1,034
1	1	5,9	1	2,43	68,26	2,33	149,79	1,162
4	0	6,6	1	2,38	85,07		149,65	1,015
1	0	5,9	1	2,24	84,56	0,77	227,37	1,676
4	0	6,2	1	1,44	60,62		171,92	0,904
1	0	6,3	1	8,04	78,95	11,91	219,26	0,966
1	0	5,3	1	3,01	65,71	0,30	244,43	0,890
1	0	6,4	1	2,19	94,75	0,51	166,31	0,797
4	1	6,8	1	3,83	78,95		189,92	5,174
5	0	7,7	2	2,17	62,65	16,15	220,85	1,478
2	0	8,6	2	3,01	86,60	29,06	246,00	1,545
5	0	8,8	2	15,08	66,22		260,75	1,730
2	0	7,6	2	24,37	110,03	0,30	196,18	1,098
5	0	8,8	2	7,13	73,35		218,75	1,055
5	0	8,6	2	3,00	4,08	40,34	295,08	0,951
2	0	7	2	3,52	5,60		209,68	1,331
2	0	7,7	2	3,39	74,37	22,50	189,48	1,931
5	0	8,6	2	1,71	82,01	0,08	200,41	1,103
5	0	8,8	2	1,91	80,48		240,50	1,086
5	1	7,1	2	1,67	143,65	0,72	204,37	0,865
5	0	7,4	2	2,08	90,67	3,81	140,06	0,958
2	0	7,2	2	6,92	93,22		185,49	3,701
2	1	7	2		89,14	2,71	164,14	1,086
5	0	7,5	2	1,30	60,11	0,72	143,92	1,223
5	1	7,3	2	2,16	142,63	1,91	159,70	1,324
5	1	8,5	2	3,34	69,28		255,92	1,120
5	1	9	2	1,62	135,50		221,57	1,395
2	0	7,5	2	2,18	50,43		187,95	1,020
2	1	7,8	2	2,09	62,15	0,92	239,47	1,159
2	0	8,6	2	2,12	95,26		207,32	0,951
2	1	7,1	2	0,97	63,67		262,02	1,375

2	0	8,2	2	2,28	77,94		205,84	1,919
2	0	7,2	2	2,85	72,33	1,12	135,31	0,936
2	0	8,8	2	2,14	75,90		234,45	2,823
2	0	7,8	2	1,56	72,84	4,21	168,38	0,939
2	1	8,8	2	4,56	92,71	1,78	239,62	1,507
2	0	7,1	2	2,67	81,50		162,63	0,740
5	0	7,9	2	2,10	60,11		181,21	1,181
5	1	8,7	2	2,43	68,77	4,37	186,37	0,814
5	1	7,8	2	1,97	61,13	0,14	153,69	1,022
5	0	7,3	2	2,97	71,31		218,17	1,331
3	0	11,4	3	3,74	74,62	8,36	269,71	1,070
6	0	13,2	3	3,57	55,01	14,84	223,51	1,005
6	1	10,7	3	9,70	83,28	4,73	276,52	1,216
3	1	10,7	3	16,20	53,23	9,39	201,70	1,145
6	0	11,6	3	20,08	63,42	2,30	190,25	2,038
6	0	11,1	3	1,39	68,77	0,27	219,28	2,072
6	0	14	3	3,95	70,30		274,93	1,514
3	0	9,2	3	3,74	64,69	9,70	240,66	1,463
6	0	9,5	3	3,99	88,12	33,67	153,32	0,895
6	0	9,2	3	2,40	62,15	10,44	149,25	1,157
6	0	9,7	3	3,64	6,37	86,64	217,78	1,520
6	0	14,3	3	2,18	68,26	0,92	217,45	1,260
6	0	11,2	3	1,60	68,77	0,05	231,08	1,500
6	0	11,4	3	2,66	94,24	5,82	270,29	1,127
3	0	10,7	3	1,71	129,38		225,61	0,955
3	1	10,7	3	2,38	110,03	1,83	178,01	0,838
3	1	11,4	3	1,92	74,37		287,55	0,708
3	0	9,5	3	1,83	74,88		136,70	1,025
3	0	12,1	3	2,20	64,69	3,37	211,29	0,922
6	1	11,6	3	1,93	85,58	3,20	186,42	0,897
3	0	9,3	3	2,06	64,69	0,68	191,27	0,797
6	0	9,6	3	3,37	123,27	11,66	124,37	1,186
6	0	11,6	3	2,01	70,80		130,74	1,434
6	0	9,9	3	1,83	114,10	0,01	166,07	1,382
3	0	12,7	3	2,10	82,01	6,32	146,16	1,147
3	1	9,2	3	2,37	83,03		210,56	1,397
3	0	15,4	3	2,56	82,01		200,24	1,485
3	0	10,8	3	2,17	101,88		164,63	1,096
5	0	8,5	2	13,24	63,42		204,27	1,249
7		6,3	7	3,13	92,20	0,69	204,06	1,973
7		6,1	7	2,23	50,94	8,83	248,02	1,203
7		6,4	7	1,56	85,07	1,99	211,445	1,721
7		6,2	7	2,40	101,37	2,02	236,52	1,140
7		6,2	7	0,87	76,41	0,18	169,18	3,368
7		6	7	2,40	67,24	2,98	216,64	1,333
7		6,2	7	1,87	71,31	3,05	238,04	2,169
7		5,7	7	3,34	106,97		179,50	1,385
7		6,3	7	0,92	83,03		250,93	1,088
7		6,2	7	2,04	83,54	1,12	143,63	1,098
7		6,5	7	2,74	84,00		218,40	1,255

7	5,7	7				227,81	1,527
8	5,6	8	1,68	88,12	1,63	258,79	1,228
8	5,5	8	1,80	148,74	1,82	371,62	1,216
8	5,2	8	1,63	55,52		221,03	1,201
8	5,6	8	1,93	64,69	5,47	174,87	1,958
8	5,3	8	0,95	76,92	0,71	184,16	1,137
8	5,2	8	0,84	113,59	0,49	177,08	1,152
8	5,4	8	1,63	61,13	6,78	294,93	0,990
8	5,4	8	5,20	77,43			1,294
8	5,4	8	1,39	84,56	0,24	373,24	1,130
8		8	1,67	103,91		335,34	1,184
8		8	1,97		1,42	309,23	1,110
8		8				269,56	

TNFa	IMC	obesidade	CA	Quadril	RCQ	PAsist	PAdiast	HAS	TpoHAS
	35,4	2				120	80	1	23
						125	75	1	10
	19,8	0				123	69	0	
	25,7	4	91	94	0,97	122	69	0	
	30,4	1	102	108	0,94	117	65	1	24
	30,3	1	102	105	0,97	118	70	1	46
	47,5	3	117	133	0,88	115	74	1	10
	26,8	4	89	96	0,93	120	70	1	12
	34,2	1	100	100	1,00	115	67	0	
	30,4	1	106	103	1,03	100	65	0	
	28,8	4	104	102	1,02	110	65	1	11
	22,8	0	83	88	0,94	100	60	1	2
	37,2	2	132	119	1,11	145	90	0	
	30,8	1	97	102	0,95	110	62	1	3
	27,6	4	106	106	1,00	116	76	0	
0,408	33,1	1	114	109	1,05	140	70	1	8
0,374	30,6	1	100	110	0,91	140	75	1	20
	32,7	1	118	117	1,01	175	80	1	30
	31,4	1	95	111	0,86	122	82	1	9
0,608	29,7	4	106	110	0,96	115	60	1	8
0,297	26,6	4	56	48	1,17	120	70	0	
2,846	26,9	4	100	102	0,98	151	68	1	10
0,274	27,3	4	95	104	0,91	125	60	1	15
0,942	24,3	0	66	92	0,72	155	98	1	4
4,517	27,4	4	98	103	0,95	105	60	1	8
0,073	21,7	0	86	100	0,86	135	80	1	21
0,942	29,6	4	102	100	1,02	160	73	1	19
	37,5	2				155	110	1	20
	32,6	1				125	83	0	
	27,0	4	105	100	1,05	110	60	0	
	27,8	4	90	103	0,87	100	60	0	
	28,8	4	89	97	0,92	140	77	1	5
	35,5	2	112	110	1,02	130	70	1	6
	26,9	4	96	90	1,07	125	75	0	
	35,3	2	109	107	1,02	120	70	1	9
	39,6	2	110	113	0,97	195	108	1	17
	40,1	3	126	114	1,11	195	100	1	16
	28,7	4	94	96	0,98	145	60	1	3
	38,0	2	121	124	0,98	131	75	1	20
	35,9	2	106	115	0,92	165	100	1	22
	28,6	4	91	104	0,88	120	70	1	9
	32,8	1	120	116	1,03	170	103	1	2
	37,2	2	115	124	0,93	100	70	1	11
	32,3	1	106	105	1,01	130	60	1	11
	33,0	1	114	102	1,12	130	85	1	20
	33,1	1	91	112	0,81	120	70	1	15
	30,3	1	102	102	1,00	140	60	1	34
	30,3	1	94	96	0,98	120	90	1	0,8
	43,7	3	127	137	0,93	132	88	1	18

1,410	30,1	1	96	106	0,91	135	75	1	10
0,040	27,1	4	88			120	70	1	5
0,909	32,4	1	103	107	0,96	119	70	1	23
0,274	24,0	0	88	94	0,94	120	63	1	11
	29,4	4	101	109	0,93	120	75	1	8
1,343	24,0	0	90	97	0,93	105	70	1	7
0,641	26,8	4	106	103	1,03	128	62	1	20
1,209	24,4	0	95	106	0,90	139	70	1	6
0,374	34,1	1	112	111	1,01	131	76	1	25
2,579	38,0	2	128	116	1,10	108	59	0	
	32,9	1	84	101	0,83	145	68	1	10
	23,1	0				150	90	1	10
	25,7	4	91			130	78	1	18
	24,4	0	82			110	75	1	4
	33,3	1	111	103	1,08	135	75	1	11
	31,2	1	97	104	0,93	110	72	1	9
	32,0	1	100	117	0,85	160	90	1	11
	28,2	4	98	100	0,98	140	78	1	5
	31,8	1	99	107	0,93	140	100	1	2
	24,4	0	86.5	92	0,94	182	95	1	20
	41,0	3	116	122	0,95	154	75	1	20
	23,8	0	86	87	0,99	100	60	0	
	32,0	1				140	75	1	14
	31,2	1	97	101	0,96	130	60	1	7
	40,5	3	126	121	1,04	135	69	1	13
	30,0	1	111	106	1,05	120	65	1	20
	27,6	4	90	105	0,86	145	80	1	5
	29,2	4	103	105	0,98	107	62	1	26
2,011	29,7	4	98	107	0,92	155	90	1	0,08
1,443	27,8	4	109	109	1,00	150	95	1	7
	29,3	4	105	119	0,88	110	70	1	21
2,813	29,5	4	97	106	0,92	114	65	0	
1,710	29,5	4	104	110	0,95	140	83	1	6
1,410	33,6	1	101	114	0,89	141	85	1	9
1,243	23,2	0	92	100	0,92	130	71	0	
0,808	20,2	0	85	96	0,89	114	60	1	10
0,708	30,0	4	103	107	0,96	110	68	0	
0,842	26,2	4	91	101	0,90	130	85	1	3
	30,7	1	109	103	1,06	168	70	1	16
1,677									

1,04
0,54
2,18
1,84

1,310

1,143

2,880
0,241
1,610

DLP	TpoDLP	Tabagismo	Tpoabst	Ativfís	GJ	CT	LDL	HDL	TG
1	3	0		1	112	162	82	44	182
1	2	1	22		114	136	81	36	95
1	7				146	165	101	45	96
0		1	16	2	153	155	98	34	114
1	7	1	30	0	68	222	127	78	87
1	12	1	30	2	66	155	100	43	60
0		0		0	101	186	118	51	84
1	0,5	0		2	42	146	79	42	123
0		0		2	132	150	93	35	112
1	2	1	17	2	184	129	81	26	111
1	11	1	22	2	94	134	79	40	74
1	2	1	7	2	163	144	73	34	183
1	1	0		2	93	166	105	42	96
1	3	0		2	76	208	145	39	119
0		1	14	0	126	190	115	42	165
1	3	1	48	0	110	128	64	45	95
0		1	35	1	126	134	63	62	45
1	4	0		0	130	193	111	55	104
1	3	0		2	93	132	63	44	127
0		1	33	2	85	109	41	57	54
1	0,5	1	23	2	146	148	79	53	78
1	10	0		0	120	144	60	48	182
1	15	0		0	104	159	64	86	45
1	2	1	6	2	88	119	41	38	199
1	2	1	3	0	69	129	70	32	136
1	16	0		0	67	138	79	50	46
1	13	0		0	46	183	123	44	78
0		1	18	0	99	147	96	37	70
1	1	1	2	0	154	125	75	32	90
1	5	1	19	2	167	189	123	41	125
0		0		2	145	171	92	57	92
0		0		0	173	189	110	68	54
1	6	0		0	146	167	97	26	218
1	5	1	30	2	124	177	90	46	204
1	9	1	20	2	162	193	105	44	219
0		1	17	0	284	188	105	63	101
1	6	0		0	159	145	74	37	172
1	14	0		2	152	141	80	51	50
1	5	0		0	76	150	81	61	38
1	3	1	5	0	174	293		41	742
1	9	1	30	0	119	151	87	42	110
1	1	0		0	180	157	100	45	58
1	7	0		0	160	128	79	38	57
1	8	1	30	0	149	128	70	38	100
1	7	1	3	1	188	176	104	44	142
1	15	0		0	85	173	105	54	70
1	9	1	3	0	123	254	179	45	151
1	0,8	1	9	0	204	167	93	52	112
1	4	1	12	0	132	202	133	32	183

1	4	0		1	141	132	52	29	257
1	10	1	28	0	110	142	83	37	108
1	6	0		1	191	262	187	43	159
1	10	0		0	60	201	130	47	121
1	8	1	14	2	212	262	161	35	330
1	6	0		2	102	139	86	40	65
1	5	1	40	2	179	165	81	70	71
1	2	1	14	0	156	204	143	51	51
1	20	1	30	2	148	112		27	464
1	4	1	4	0	144	289	170	80	196
0	6	0		2	169	170	101	57	58
1	10	0		1	165	183	96	73	71
0		1	4	1	245	126	63	53	50
1	7	0		0	110	124	69	41	70
1	1	0		1	87	223	152	41	149
1	8	0		0	143	243	161	47	176
1	8	0		1	48	246	161	54	154
1	11	1	17	1	148	191	117	60	72
0		0		0	133	163	113	34	80
1	2	0		2	115	152	74	65	63
1	9	0		0	41	169	84	42	213
1	0,42	1	25	0	311	255	194	48	66
1	13	0		0	152	224	132	60	38
1	5	1	3	1	287	225	164	32	147
1	5	1	32	0	126	133	79	25	141
1	20	0		0	133	138	76	23	196
1	4	1	1	0	203	142	95	34	64
1	5	1	4	0	227	162	90	56	80
0		1	10	0	477	183	115	41	137
1	2	0		0	375	273	199	36	192
0		1	20	1	306	176	103	61	62
1	5	1	21	0	166	217	118	38	305
0		0		0	288	190	118	46	128
1	4	0		0	226	214	155	40	97
1	0,08	0		0	88	227	154	59	68
0		0		0	232	201	117	58	
1	9	0		0	321	242	164	62	77
0		0		0	79	177	118	38	105
1	1	0		2	103	135	56	60	97
					90	164	76	50	192
					84	154	91	48	77
					94	185	107	43	175
					89	206	84	38	420
					95	268	158	57	263
					60				
					77	235	173	43	91
					121	175	119	31	125

111	138	40	26	453
87	214	109	93	62
90	199	130	38	153
93	155	91	42	108
93	249	165	66	91
95	229	156	49	122
83	188	129	44	74

uréia	cr	Clcr	Estatina	SU	IECA	BRA	insulina	MTF	inib_DDP4
38	0,71	107	1	0	0	1	0	1	1
36	1,04	76	1	0	1	0	1	1	0
27	0,74	99	1	1	1	0	1	1	0
48	1,01	80	0	1	0	0	0	1	1
31	0,81	82	1	0	0	1	1	1	0
34	1,36	65	1	0	1	0	1	1	0
34	0,79	77	0	0	1	0	0	1	0
33	0,91	94	1	1	1	0	0	1	0
31	1,03	86	0	0	0	0	1	1	0
25	0,98	87	1	1	0	0	0	1	1
44	0,84	93	1	1	1	0	1	1	0
40	0,84	81	1	1	0	0	0	1	0
28	0,88	101	1	1	0	0	0	1	0
26	0,66	107	1	0	0	1	0	1	0
30	0,77	93	0	0	0	0	0	1	0
32	1,1	69	1	1	0	1	0	1	0
33	0,68	96	0	0	1	0	0	1	0
26	0,79	93	1	1	0	1	0	1	0
20	0,79	79	1	1	0	1	0	1	0
44	1,03	76	1	1	1	0	0	1	0
37	1,03	89	1	1	0	0	0	1	1
	0,85	72	1	0	1	0	1	1	0
41	0,93	61	1	0	1	0	1	1	0
43	0,63	100	1	0	0	1	0	1	0
35	0,98	74	1	0	0	0	1	1	0
43	0,79	76	1	0	1	0	1	1	0
36	1	69	1	0	0	1	1	1	0
31	0,75	89	0	1	1	1	1	1	0
22	0,86	103	1	0	0	0	1	1	0
32	0,87	100	1	1	0	1	1	1	0
24	0,94	83	0	0	0	0	1	1	0
35	0,87	68	0	0	1	0	1	1	0
61	1	63	1	0	0	1	1	1	0
22	0,65	99	1	0	0	0	1	1	0
21	0,81	95	1	1	0	1	0	1	0
30	0,73	86	0	0	1	0	1	1	0
25	0,87	77	1	0	0	1	1	1	0
32	0,66	99	1	1	0	1	1	1	0
41	0,92	79	1	0	1	0	1	1	0
57	1	65	0	0	0	1	0	1	0
32	1,04	60	1	1	1	0	1	1	0
46	0,78	100	1	1	1	0	0	1	0
52	1,16	73	1	1	1	0	0	1	0
34	0,77	80	1	0	0	1	1	1	0
20	0,78	101	1	0	0	1	1	1	0
26	0,74	96	1	0	0	1	1	0	0
45	0,98	70	1	0	1	0	0	1	0
27	0,8	80	1	1	1	0	1	1	0
29	0,68	103	1	1	0	1	0	1	0

27	1	95	1	1	0	1	1	1	0
33	0,3	126	1	1	1	0	0	1	0
36	0,92	67	1	0	1	0	0	1	0
42	0,99	61	1	0	1	0	1	1	0
23	1,08	92	1	1	0	1	0	1	0
37	0,69	90	1	0	1	0	1	1	0
40	1,08	72	1	0	0	1	1	0	0
48	0,96	66	1	0	0	1	1	1	0
37	0,8	82	1	1	0	1	0	1	0
44	0,64	98	1	0	1	0	0	1	0
23	0,78	82	0	0	1	0	1	1	0
32	0,87	72	1	0	1	0	1	1	0
33	0,53	108	0	0	0	1	1	1	0
20	0,68	116	1	0	0	1	1	1	0
29	0,59	110	1	0	1	0	1	1	0
22	0,54	104	1	0	1	0	1	1	0
25	0,88	86	1	0	1	1	1	1	0
37	0,87	80	1	0	1	0	1	1	0
31	1,01	68	0	0	0	1	1	1	0
29	0,96	80	1	0	0	1	1	1	0
42	0,83	79	1	0	1	1	1	1	0
27	0,6	109	1	0	0	0	1	1	0
39	0,79	80	1	0	0	1	1	1	0
34	0,81	91	1	0	1	0	1	1	0
41	1,11	69	1	0	0	1	1	1	0
26	0,93	68	1	0	1	0	1	1	0
25	0,78	83	1	0	0	1	1	1	0
21	0,68	123	1	0	1	0	1	1	0
32	0,99	78	0	0	0	0	1	0	0
43	1,26	65	1	0	0	1	1	1	0
26	0,66	110	0	0	0	0	1	1	0
31	0,56	101	1	0	0	0	1	1	0
29	0,91	78	0	0	0	1	1	1	0
24	0,81	86	1	0	0	1	1	1	0
29	0,8	93	1	0	0	0	1	0	0
27	0,8	77	0	1	0	0	1	0	0
37	0,9	94	0	1	0	0	1	1	0
28	0,62	138	0	0	1	0	1	1	0
30	0,9	70	1	0	0	0	1	1	0

AAS	b_bloq	furosemida	HCTZ	bloq canais Ca	hidralazina
0	0	0	1	0	0
1	0	0	0	1	0
1	0	0	0	0	0
1	0	0	0	0	0
1	0	0	1	0	0
1	0	0	1	1	0
1	0	0	1	1	0
0	0	0	0	0	0
1	0	0	0	0	0
1	0	0	0	0	0
1	0	0	1	0	0
0	0	0	0	0	0
0	0	0	0	0	0
1	0	0	0	0	0
0	0	0	0	0	0
1	1	1	0	1	0
0	1	1	0	1	0
1	0	1	0	1	1
1	0	0	0	0	0
1	1	0	0	0	0
1	0	0	0	0	0
1	1	0	1	0	0
1	0	0	1	0	0
0	0	0	1	0	0
0	0	0	0	0	0
0	0	0	1	0	0
1	0	1	0	0	1
0	1	0	1	1	0
0	0	0	0	0	0
1	0	0	0	0	0
0	0	0	0	0	0
0	0	0	1	0	0
0	1	0	1	0	0
0	1	0	0	0	0
1	1	0	1	0	0
1	0	0	0	1	0
0	0	0	0	0	0
1	1	0	0	1	0
1	0	0	1	1	0
0	1	1	0	1	0
1	1	0	0	0	0
1	1	0	0	0	0
0	1	1	0	0	0
1	1	0	0	0	0
1	1	0	0	1	0
1	0	0	1	0	0
1	0	0	1	0	0
0	0	0	0	0	0
0	1	1	0	0	0

0	0	0	1	1	0
1	0	0	1	0	0
0	0	0	1	1	0
1	1	0	1	1	0
1	1	0	1	1	0
0	0	0	0	0	0
1	0	0	1	1	0
1	0	0	1	0	0
1	1	1	0	0	0
0	0	0	0	0	0
1	0	0	1	1	1
1	0	0	0	1	0
1	0	0	1	0	0
1	1	0	1	0	0
0	1	0	1	0	0
1	1	0	1	1	0
1	0	0	1	1	0
1	0	0	1	1	0
1	0	0	1	1	0
1	1	0	1	1	0
1	0	0	0	1	1
0	0	0	0	0	0
1	1	0	0	0	0
0	0	0	1	0	0
1	0	0	1	1	0
1	1	0	1	0	0
1	1	0	1	0	0
0	0	0	0	0	0
0	0	0	0	0	0
0	1	0	0	1	0
0	0	0	0	0	0
0	0	0	0	0	0
0	0	0	0	0	0
0	0	0	1	1	0
1	0	0	0	0	0
1	1	0	0	0	0
0	0	0	0	0	0
0	1	0	0	0	0
1	0	0	1	1	0
